# miRNA‐765 mediates multidrug resistance via targeting BATF2 in gastric cancer cells

**DOI:** 10.1002/2211-5463.12838

**Published:** 2020-04-18

**Authors:** Wan Lin, Yu Miao, Xiangkun Meng, Ying Huang, Wanli Zhao, Jigang Ruan

**Affiliations:** ^1^ Department of Gastroenterology General Hospital of Ningxia Medical University Yinchuan China; ^2^ Department of Anesthesiology General Hospital of Ningxia Medical University Yinchuan China

**Keywords:** basic leucine zipper ATF‐like transcription factor 2, gastric cancer, miRNA‐765, multidrug resistance

## Abstract

Elucidation of the mechanisms underlying multidrug resistance (MDR) is required to ensure the efficacy of chemotherapy against gastric cancer (GC). To investigate this issue, here we identified that microRNA‐765 (miRNA‐765) is up‐regulated both in MDR GC cell lines and in specimens from patients who are not responding to chemotherapy. In addition, down‐regulation of miRNA‐765 increased the sensitivity of GC cells to anticancer drugs, whereas its overexpression had the opposite effect. Moreover, miRNA‐765 suppressed drug‐induced apoptosis and positively regulated the expression of MDR‐related genes. Finally, we showed that the basic leucine zipper ATF‐like transcription factor 2, a tumor suppressor gene, is the functional target of miRNA‐765. In summary, these results suggest that miRNA‐765 may promote MDR via basic leucine zipper ATF‐like transcription factor 2 in GC cells.

## Graphical abstract

microRNA‐765 (miRNA‐765) is upregulated in multidrug‐resistant (MDR) gastric cancer (GC) samples. miRNA‐765 binds to the 3′ UTR of basic leucine zipper ATF‐like transcription factor 2 mRNA and inhibits basic leucine zipper ATF‐like transcription factor 2 expression, thereby suppressing drug‐induced apoptosis and positively regulating the expression of MDR‐related genes, characterized as increased viability of the tumor cells and decreased sensitivity to chemotherapy.

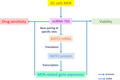

**Abbreviations**
BATF2: basic leucine zipper ATF‐like transcription factor 2
GAPDH: glyceraldehyde‐3‐phosphate dehydrogenase
GC: gastric cancer
IC_50_: half maximal inhibitory concentration
LRP: lung resistance protein
MDR: multidrug resistance
miRNA: microRNA
miRNA‐765: microRNA‐765
MTT: 3‐(4,5‐dimethylthiazol‐2‐yl)‐2,5‐diphenyl‐tetrazolium bromide
Mut: mutated
P‐gp: P‐glycoprotein
qRT‐PCR: quantitative RT‐PCR
VCR: vincristine
WT: wild‐type

In China, gastric cancer (GC) is a common malignant disease of the digestive system, with the second highest incidence and the third highest mortality rate [1]. It is reported that the 5‐year survival rate of patients with advanced GC is only 30–40%. Chemotherapy is commonly used for the comprehensive clinical treatment of GC. Nevertheless, the efficacy of chemotherapy is markedly compromised by the development of multidrug resistance (MDR) [2, 3]. Multiple mechanisms underlying MDR, including limitation of drug uptake, drug metabolism, augmented efflux, augmented DNA damage repair, inhibition of apoptosis and other unknown cell functions, may explain MDR [4, 5]. Nonetheless, the detailed molecular mechanisms of MDR have not been fully elucidated.

MicroRNAs (miRNA) are small noncoding RNAs with multiple regulatory functions, which are related to a broad range of physiological and pathological processes, including the development of cancer and progression. Usually, miRNAs target the 3′ UTRs of certain mRNAs to down‐regulate the expression of genes. Recently, noncoding RNAs, such as miRNAs, have been reported to participate in the development of MDR [6, 7]. For instance, miRNA‐23b‐3p can render GC cells more sensitive to chemical agents by controlling the expression of HMGB2 and ATG12 [8]. Up‐regulation of miRNA‐181b or miRNA‐497 is capable of improving the sensitivity of GC cells to chemotherapy by modulating the important antiapoptotic gene *Bcl‐2* [9, 10]. miR‑765 is reportedly abnormally expressed and contributes to the tumorigenesis of many different types of human cancer, such as esophageal squamous cell carcinoma [11], osteosarcoma [12], and hepatocellular carcinoma [13]. Moreover, a previous study demonstrated that miRNA‑765 participates in primary breast carcinoma by regulating the epithelial membrane protein 3 [14]. Another study revealed that miR‑765 serves an oncogenic role in multiple myeloma progression by directly targeting SOX6 [15]. MicroRNA‐765 (miRNA‐765) was also reported as a key mediator for inhibiting growth, migration and invasion in fulvestrant‐treated prostate cancer. All of these suggested that miR‐765 was involved in the development of several types of cancer. However, the expression pattern, the specific roles and the underlying mechanism of miR‑765 in the MDR of GC remain largely unknown.

The aim of this study was to investigate the roles of miRNA‑765 in GC MDR. The expression of miRNA‑765 was detected in GC tumor tissue samples and the vincristine (VCR)‐resistant GC cell line SGC7901 (SGC7901/VCR). Furthermore, SGC‐7901/VCR cells with a stable down‐regulation of miRNA‑765 and SGC‐7901 cells with a stable overexpression of miRNA‑765 were constructed. Subsequently, changes in the half maximal inhibitory concentration (IC_50_), clone number, cell cycle and apoptosis were investigated. Moreover, we investigated the impact of miRNA‑765 on target gene basic leucine zipper ATF‐like transcription factor 2 (BATF2) and the expression of genes associated with MDR to identify the underlying mechanism. Data obtained in this study may aid the elucidation of the functional roles of miR‑765 in MDR of GC.

## Materials and methods

### Ethical statement

The experimental protocols were approved by the Ethics Committee of General Hospital of Ningxia Medical University and performed according to the guidelines of the 1975 Declaration of Helsinki. All participants involved in this study provided written informed consent.

### Specimen collection

A total of 28 MDR GC tissues and 16 nontreatment GC tissues were collected from General Hospital of Ningxia Medical University. The pathological diagnosis of each specimen was evaluated by a pathologist. The clinicopathological features were obtained from the medical records of patients with GC (Table 1). All tissue specimens were stored at −80 °C until use.

**Table 1:** The characteristics of the cases in this study.

Characteristics	No. of cases (*N* = 44)
Sex	
Male	23
Female	21
Age (years)	
<65	18
≥65	26
Tumor size (cm)	
<3.5	21
≥3.5	23
TNM stage	
I–II	17
III–IV	27
Carcinoembryonic antigen (ng·mL^−1^)	
<5	15
≥5	29
CA724 (U·mL^−1^)	
<8.2	17
≥8.2	27
Histological grade	
Well‐moderate	19
Poor‐signet	25

### Cell culture

The SGC7901 (human gastric adenocarcinoma cell line) was purchased from the Cell Bank of Chinese Academy of Science (Shanghai, China). The SGC7901/VCR (MDR variants) were constructed and cultured in our laboratory. Both cell lines were cultured in RPMI 1640 medium (Thermo Fisher Scientific, Grand Island, NY, USA) supplemented with 10% FBS (Invitrogen, Carlsbad, CA, USA) at 37 °C with 5% CO_2_.

### Cell transfection

The miRNA‐765 mimic and/or inhibitor and matching control were obtained from RiboBio Company (Guangzhou, China). The miRNA‐765 mimic/inhibitor or the matching control (150 nm) was transiently transfected into GC cells using RNAiMAX (Invitrogen). The cells were collected and treated 48 h after transfection.

### RNA isolation and quantitative RT‐PCR

miRNAs were extracted using the miRNA Extraction Kit obtained from Tiangen (Beijing, China). After adding the Poly(A) first, RNA (1 μg) containing miRNAs was reversely transcribed into cDNA. We obtained primers of U6 and miRNA‐765 from Sangon Biotech (Shanghai, China) to detect the level of miRNA‐765. Moreover, total RNA was isolated using the TRIzol Kit (Invitrogen). Subsequently, total RNA was reversely transcribed into cDNA using a Promega reverse transcription kit (Madison, WI, USA) for the detection of BATF2 expression. The miRNA expression or BATF2 expression was analyzed using SYBR Premix Ex Taq from Takara (Dalian, China) in an ABI QS6 system (ABI). U6 or glyceraldehyde‐3‐phosphate dehydrogenase (GAPDH) was used as endogenous control. The quantitative RT‐PCR (qRT‐PCR) primers were as follows: BATF2‐F, 5‐CACCAGCAGCACGAGTCTC‐3 and BATF2‐R, 5‐TGTGCGAGGCAAACAGGAG‐3; GAPDH‐F, 5‐GGTGGTCTCCTCTGACTTCAACA‐3 and GAPDH‐R, 5‐GTTGCTGTAGCCAAATTCGTTGT‐3.

### Western blotting

After transfection (48 h), cells were harvested and lysed for the extraction of total protein. Primary antibodies against BATF2, P‐glycoprotein (P‐gp), lung resistance protein (LRP) and MDR‐associated protein 1 (MRP1) were purchased from Abcam (Cambridge, MA, USA). Primary antibodies against GAPDH (#5174) were purchased from Cell Signaling Technology (Danvers, MA, USA). Secondary antibodies containing anti‐rabbit horseradish peroxidase and anti‐mouse horseradish peroxidase were obtained from Santa Cruz Biotechnology (Santa Cruz, CA, USA).

### Drug sensitivity assay *in vitro*

Cells were seeded into 96‐well plates (8000 cells/well) and cultured overnight. Subsequently, the cells were incubated for 24 h with freshly prepared chemotherapeutic drugs, VCR. For the 3‐(4,5‐dimethylthiazol‐2‐yl)‐2,5‐diphenyl‐tetrazolium bromide (MTT) analysis, cells were incubated with MTT for another 4 h in each well. The absorbance at 490 nm was recorded using a microplate reader, and the relative survival curve was performed to evaluate the IC_50_ of each drug.

### Flow cytometric analysis of apoptosis

After transfection, SGC7901 or SGC7901/VCR cells (250 000 cells/dish) were seeded in a 10‐cm dish. Subsequently (48 h), when the cell density reached 80% confluency, they were harvested, washed in PBS and stained with Annexin V and 7‐aminoactinomycin D for 30 min. The samples were then analyzed using a flow cytometer.

### Luciferase reporter assays

The wild‐type (WT) or mutated (Mut) human BATF2 3′ UTR sequence including potential binding sites was amplified and cloned into the pGL3‐Basic vector (Promega). A mixture of 20 ng pGL3‐Basic‐BATF2 and 150 nm miRNA‐765 mimic was transiently transfected together into 293T cells. After transfection (48 h), firefly luciferase activity and relative Renilla expression were measured using the Dual‐Luciferase Reporter Assay System (Promega).

### Statistical analysis

All statistical analyses were performed using the prism 6 software (La Jolla, CA, USA). All data are presented as the mean ± standard deviation. Student’s *t*‐test was used for the analysis of differences. A *P*‐value <0.05 denoted statistically significant differences between groups. All experiments were carried out independently three times.

## Results

### miRNA‐765 increases in nonresponding GC tissue specimens and drug‐resistant GC cells

**Fig. 1:**
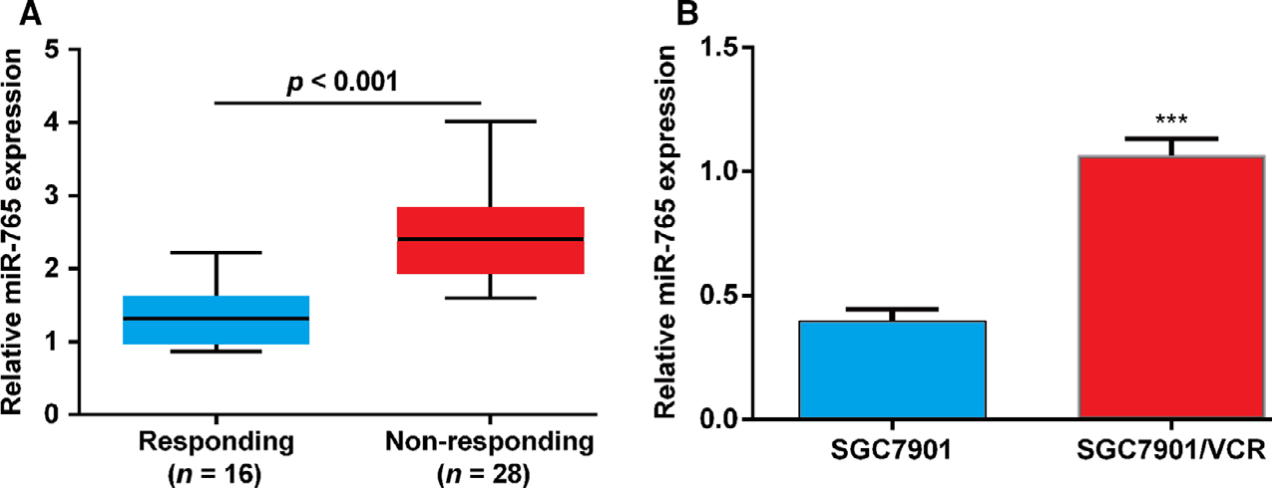
The expression of miRNA‐765 in GC tissues and drug‐resistant GC cells. (A) The expression of miRNA‐765 in tumor tissues obtained from responding patients (*n* = 16) was significantly lower than that observed in tumor tissues from nonresponding patients (*n* = 28). (B) The expression of miRNA‐765 in SGC7901/VCR and SGC7901 cells was detected using qRT‐PCR. The error bars indicate standard deviation. *t*‐Test was used for the analysis of differences. The experiments were carried out independently three times. ***P < 0.005.

The expression level of miRNA‐765 was detected in tissue samples obtained from patients with GC who were not responding to chemotherapy and compared with that recorded in samples from patients responding to chemotherapy. The purpose of this investigation was to determine whether miRNA‐765 is related to the development of MDR in GC. The qRT‐PCR data revealed that miRNA‐765 was significantly up‐regulated in nonresponding tumor tissues versus responding tumor tissues (Fig. 1A). In addition, the expression of miRNA‐765 was detected in the MDR GC cell lines VCR‐resistant SGC7901 (SGC7901/VCR) and their parental cell line SGC7901. The results of our study revealed that miRNA‐765 was augmented in SGC7901/VCR cells relative to SGC7901 cells (Fig. 1B). Collectively, these data implied that miRNA‐765 may be associated with the development of MDR in GC cells.

### Suppression of miRNA‐765 enhances the sensitivity of GC cells to anticancer drugs

**Fig. 2:**
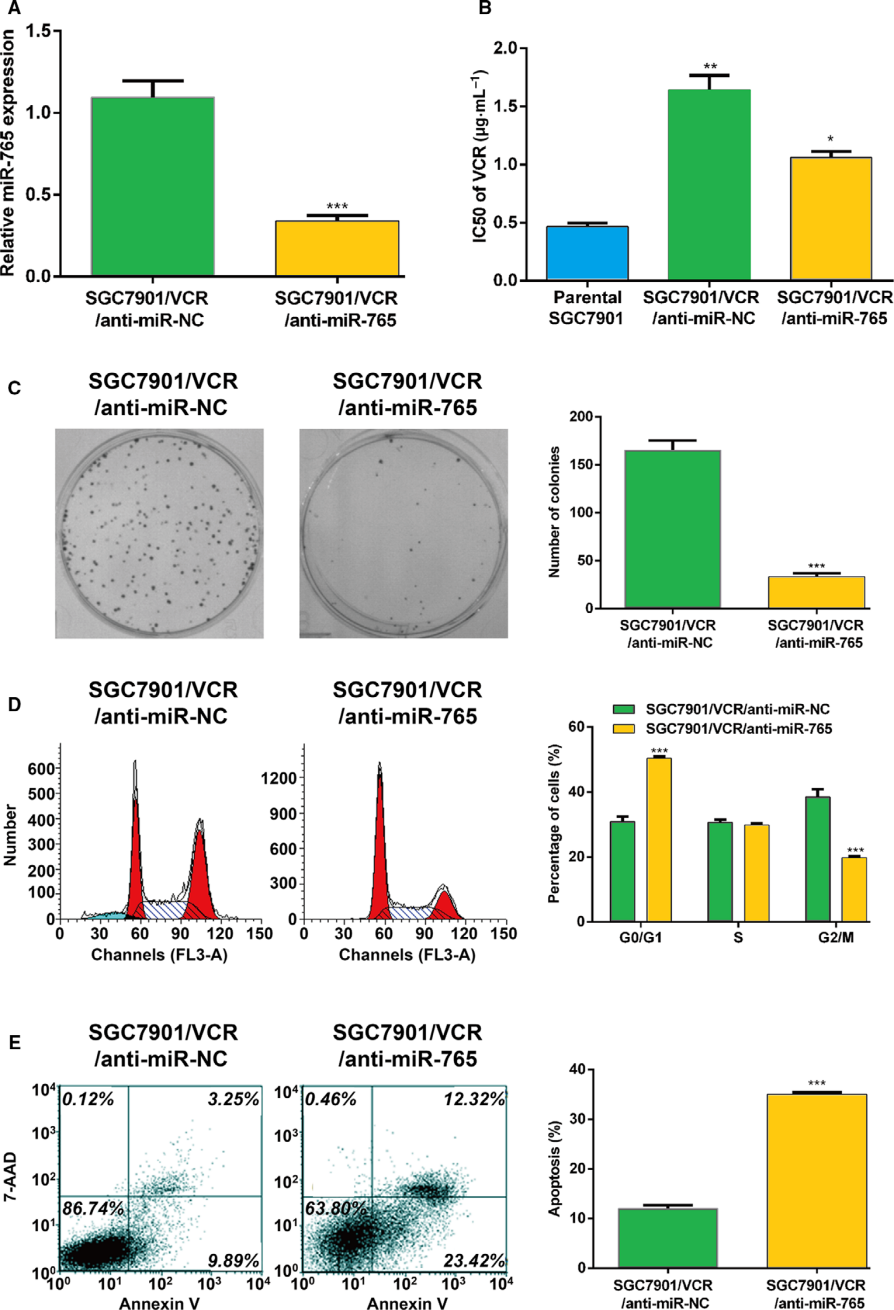
Suppression of miRNA‐765 enhances the sensitivity of GC cells to anticancer drugs. (A) qRT‐PCR analyzed the expression of miRNA‐765 after transfection of anti‐miRNA‐765 into SGC7901/VCR cells. (B) IC_50_ values for VCR after transfection of anti‐miRNA‐765 into SGC7901/VCR cells. (C) Representative colony‐forming assay images of SGC7901/VCR cells after transfection of anti‐miRNA‐765 and quantifications of colony numbers after crystal violet staining. (D) Flow cytometry was performed to assess the cell cycle distribution of SGC7901/VCR cells stained with Propidium Iodide (PI) after transfection with anti‐miRNA‐765. A representative result is shown. Red: cells at G0/G1 or G2/M phase; cyan: cell debris; dashed lines: cells at S phase. (E) Flow cytometry was used to assess the percentage of apoptotic SGC7901/VCR cells after transfection with anti‐miRNA‐765. Representative scatterplots are shown. The error bars indicate standard deviation. *t*‐Test was used for the analysis of differences. The experiments were carried out independently three times. ***P < 0.005; **P < 0.01; *P < 0.05. 7‐AAD, 7‐aminoactinomycin D.

SGC7901/VCR cells expressing lower levels of miRNA‐765 versus the control cells were transfected with a particular miRNA‐765 inhibitor to examine whether miRNA‐765 plays a direct role in the development of MDR in GC MDR (Fig. 2A). Subsequently, the effect of miRNA‐765 down‐regulation on MDR was examined in SGC7901/VCR cells. MTT assays were performed to measure the IC_50_ and sensitivity of SGC7901/VCR cells. These results revealed that miRNA‐765 deficiency in SGC7901/VCR cells led to an improved sensitivity to VCR, as indicated by diminished IC_50_ values (Fig. 2B). The colony‐forming assay confirmed these results (Fig. 2C). Furthermore, we evaluated the cells in specific phases using flow cytometry to determine whether miRNA‐765 deficiency reverses MDR by affecting the cell cycle. Interestingly, the cell counts in the G0/G1 phase were significantly increased, whereas those in the G2/M phase were decreased after transfection of SGC7901/VCR cells with anti‐miRNA‐765 (Fig. 2D). The efficacy of chemotherapeutic drugs is influenced by alterations in drug‐induced apoptosis. Therefore, the effect of miRNA‐765 on the apoptosis of SGC7901/VCR cells was evaluated through flow cytometry. The data showed that miRNA‐765 silencing significantly increased the rate of apoptosis compared with control (Fig. 2E). In summary, these results suggested that suppression of miRNA‐765 enhances the sensitivity of GC cells to anticancer drugs by altering the cell cycle and apoptosis.

### Overexpression of miRNA‐765 decreases the sensitivity of GC cells to anticancer drugs

**Fig. 3:**
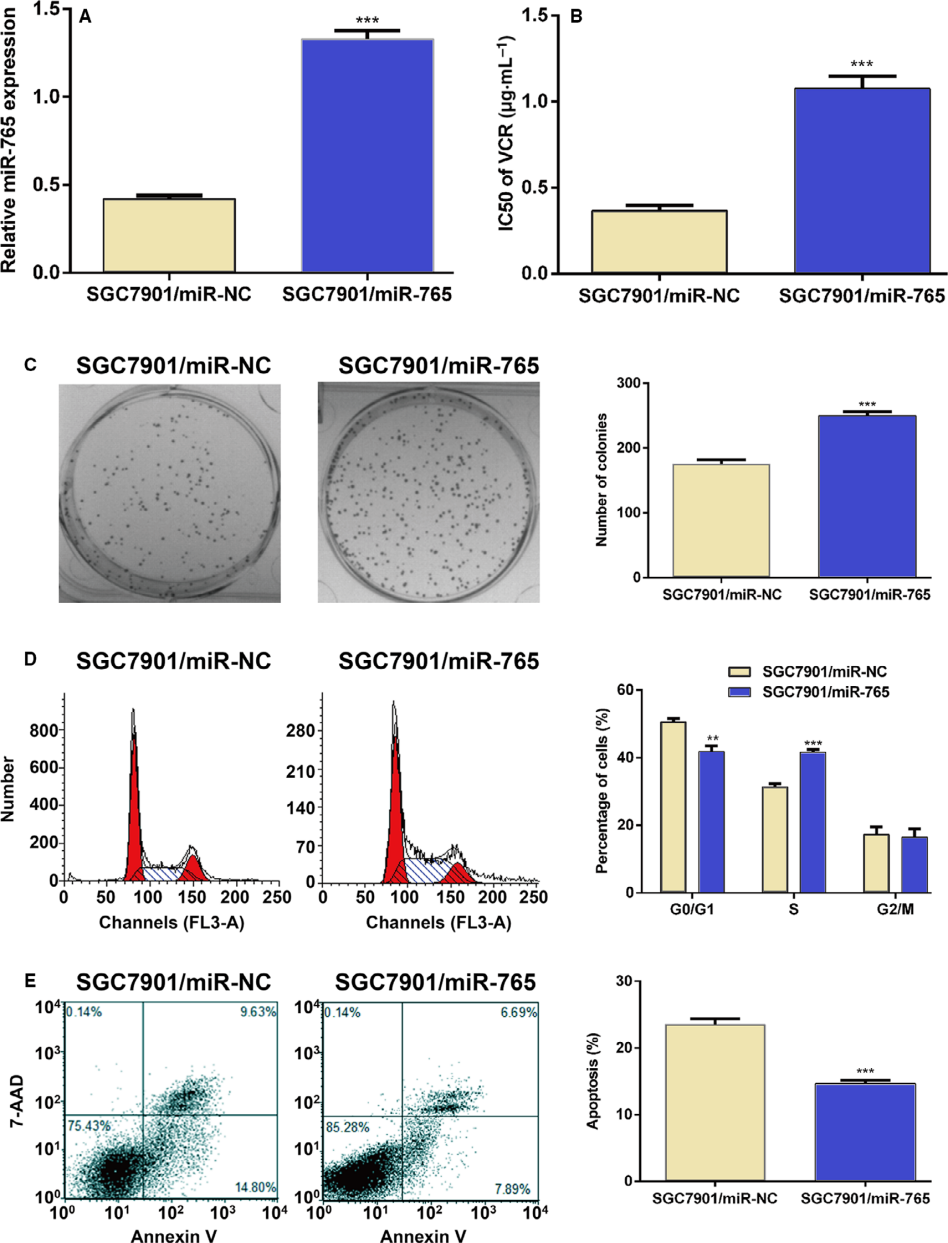
Overexpression of miRNA‐765 decreases the sensitivity of GC cells to anticancer drugs. (A) qRT‐PCR analyzed the expression of miRNA‐765 after transfection of miRNA‐765 into SGC7901 cells. (B) IC_50_ values for VCR after transfection of miRNA‐765 into SGC7901 cells. (C) Representative colony‐forming assay images of SGC7901 cells after transfection of miRNA‐765 and quantifications of colony numbers after crystal violet staining. (D) Flow cytometry was performed to assess the cell cycle distribution of SGC7901 cells stained with PI after transfection with miRNA‐765. A representative result is shown. Red: cells at G0/G1 or G2/M phase; cyan: cell debris; dashed lines: cells at S phase. (E) Flow cytometry was used to assess the percentage of apoptotic SGC7901 cells after transfection with miRNA‐765. Representative scatterplots are shown. The error bars indicate standard deviation. *t*‐Test was used for the analysis of differences. The experiments were carried out independently three times. ***P < 0.005; **P < 0.01.

To further confirm the effect of miRNA‐765 on sensitivity of GC cells, we transfected a specific miRNA‐765 mimic into SGC7901 cells to overexpress miRNA‐765. As expected, SGC7901 cells transfected with the miRNA‐765 mimic had an ectopic expression of miRNA‐765 compared with control (Fig. 3A). The results of the MTT assay showed that overexpression of miRNA‐765 in SGC7901 cells markedly decreased their sensitivity to VCR, as evidenced by the increased IC_50_ values (Fig. 3B). The colony‐forming assay also displayed an increased clone number after overexpression of miRNA‐765 versus control (Fig. 3C), confirming the decreased sensitivity. Furthermore, we analyzed the cell cycle and apoptosis using flow cytometry. The cell cycle data revealed that the number of cells in the G0/G1 phase was reduced, whereas that of cells in the S phase was increased after overexpression of miRNA‐765 (Fig. 3D). The number of apoptotic cells was significantly reduced in SGC7901 cells transfected with the miRNA‐765 mimic, as shown by the 7‐aminoactinomycin D–Annexin V analysis (Fig. 3E). Collectively, these data further confirm the important role of miRNA‐765 in determining sensitivity to chemotherapeutic drugs.

### miRNA‐765 controls MDR by targeting BATF2

**Fig. 4:**
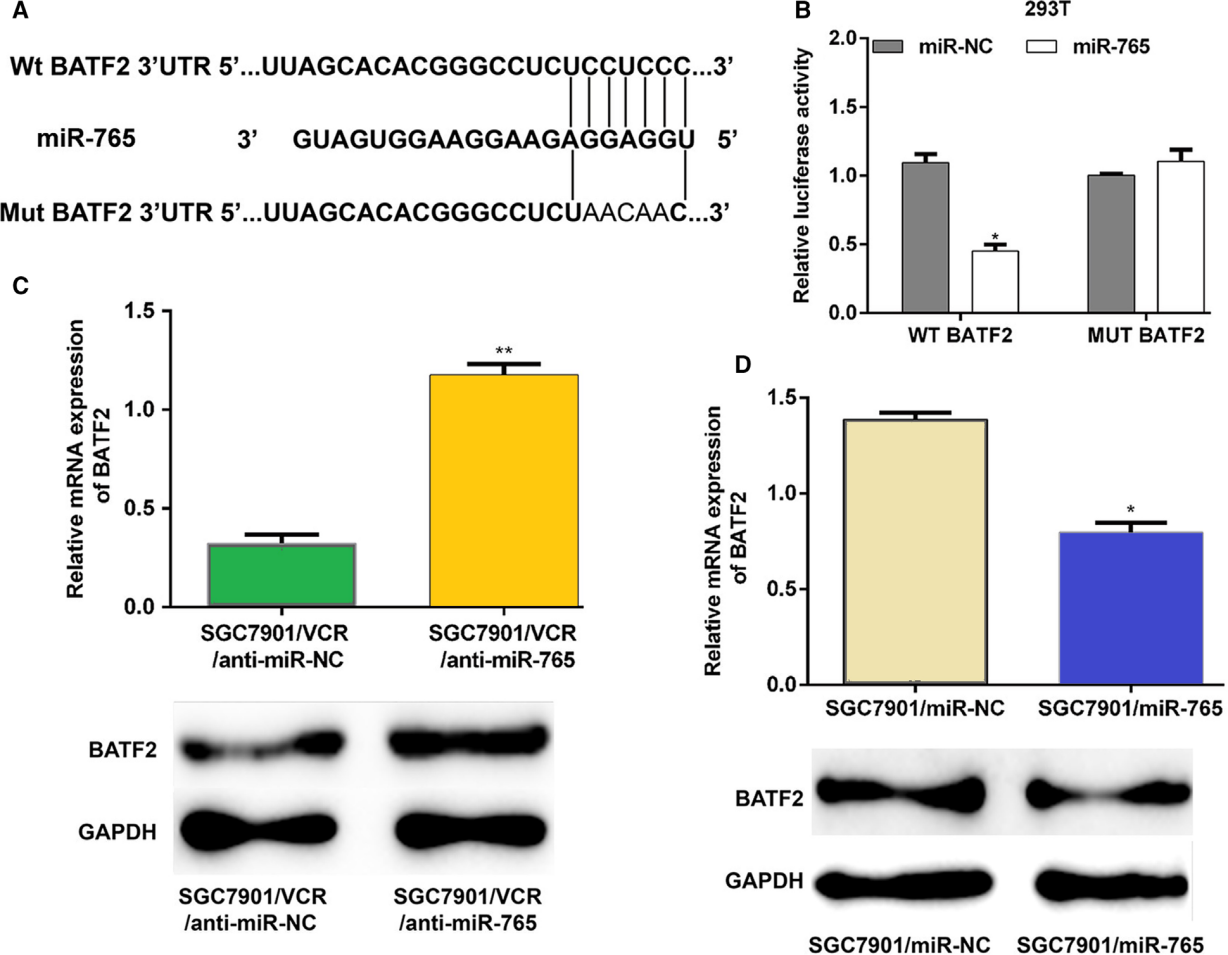
miRNA‐765 targets BATF2. (A) The miRNA‐765 target site predicted in the 3′ UTR of BATF2 mRNA and its mutant are shown. (B) WT BATF2 3′ UTR or the Mut luciferase constructed and miRNA‐765 or the control mimic were cotransfected into 293T cells for 48 h. Subsequently, luciferase activity assay was performed. (C) Anti‐miRNA‐765 was transfected into SGC7901/VCR cells. The mRNA and protein expression levels of BATF2 were measured 48 h after transfection. (D) The miRNA‐765 mimic was transfected into SGC7901 cells. The mRNA and protein expression levels of BATF2 were detected 48 h after transfection. The error bars indicate standard deviation. *t*‐Test was used for the analysis of differences. The experiments were carried out independently three times. **P < 0.01; *P < 0.05.

To determine the mechanisms underlying miR‑765 activity in MDR cells, we performed bioinformatics analysis to search for potential targets of miR‑765. BATF2 was selected for further experimental validation because BATF2 is involved in the MDR of human cancer [16, 17]. As shown in Fig. 4A, BATF2 was identified as one of the targets of miRNA‐765. The luciferase activity assay was performed in 293T cells to confirm that BATF2 is targeted by miRNA‐765. The WT or the mutant (Mut) BATF2 3′ UTR luciferase reporter plasmid was cotransfected with the control mimic or miRNA‐765 mimic. The cotransfection of the miRNA‐765 mimic with the WT luciferase reporter plasmid in 293T cells displayed significantly reduced luciferase activity compared with the control, whereas that of Mut was not altered (Fig. 4B). The levels of BATF2 mRNA and protein were significantly increased after transfection of anti‐miRNA‐765 into SGC7901/VCR cells (Fig. 4C), whereas transfection of the miRNA‐765 mimic into SGC7901 cells obviously decreased those levels (Fig. 4D). In conclusion, these data suggested that BATF2 is the target of miRNA‐765 in GC cells.

### miRNA‐765 functions by targeting MDR‐related genes

**Fig. 5:**
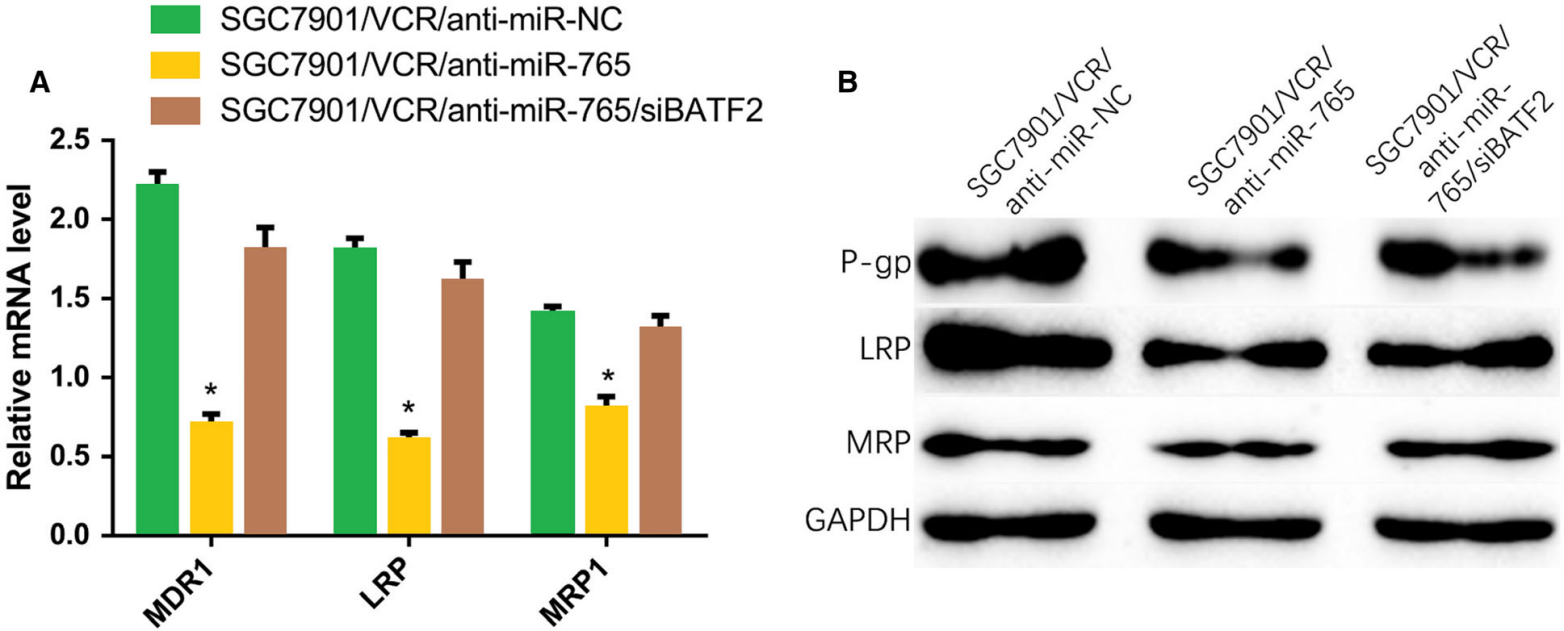
miRNA‐765 functions by targeting MDR‐related genes. (A) qRT‐PCR analysis of the expression of *MDR1*, *LRP* and *MRP1* 48 h after transfection of anti‐miRNA‐765 in SGC7901/VCR cells. (B) The protein levels of P‐gp, LRP and MRP were detected 48 h after transfection of anti‐miRNA‐765 into SGC7901/VCR cells. The error bars indicate standard deviation. *t*‐Test was used for the analysis of differences. The experiments were carried out independently three times. *P < 0.05.

The expression of MDR‐related genes was detected to further explore the mechanism through which anti‐miRNA‐765 induces the reversal of MDR in SGC7901/VCR cells. The qRT‐PCR data showed that the expression of MDR‐related genes (e.g., *MDR1*, *LRP* and *MRP1*) after transfection with anti‐miRNA‐765 was lower than that observed in SGC7901/VCR cells transfected with control, and if the cell was cotransfected with an miRNA‐765 and siBATF2, the expression of MDR‐related genes was stimulated again (Fig. 5A). Similarly, the immunoblotting assay revealed that the protein levels of P‐gp, LRP and MRP1 were decreased after suppressing miRNA‐765 in SGC7901/VCR cells, but bounced up in the presence of siBATF2 (Fig. 5B). These results established that miRNA‐765 regulates MDR by targeting P‐gp, LRP and MRP1, which have vital functions in MDR.

## Discussion

GC is the third leading cause of cancer mortality worldwide due to the low rate of early diagnosis [18]. Patients with advanced GC and metastasis miss their best chance for curative treatment (i.e., surgery); hence chemotherapy is the preferred treatment option. However, MDR is one of the most common causes of treatment failure in patients with GC who are receiving chemotherapy. The mechanisms underlying MDR have been comprehensively investigated; nevertheless, the critical factors involved in this process remain unclear. The SGC7901/VCR cell line, which was derived from the human GC cell line SGC7901 after selection with VCR (an inducing drug), has been broadly engaged as a model for studying the development of MDR in GC *in vitro*. Recently, using this model, many studies have found that numerous miRNAs, including miRNA‐1299, miRNA‐508‐5p, and miRNA‐27b, are involved in the development of MDR in GC [19, 20, 21, 22]. In this study, we identified that miRNA‐765, which is related to primary breast carcinoma [14], was increased in GC tissue from nonresponding patients and drug‐resistant GC cells SGC7901/VCR, suggesting its role in MDR. Further investigations revealed that miRNA‐765 exerted MDR by regulating BATF2‐ and MDR‐related genes, such as *MDR1*, *LRP* and *MRP1*. These findings suggest that miRNA‐765 may be potentially applied in the prediction of drug resistance and clinical treatment in the future.

A defect in drug‐induced apoptosis is one of the main causes of MDR. Previous studies revealed that alterations in the apoptosis and survival signaling pathways, including the Bcl‐2, Bax and AKT pathways, affected the efficacy of chemotherapeutic agents [23]. This study demonstrated that down‐regulation of miRNA‐765 promoted the drug‐induced apoptosis of SGC7901/VCR cells, whereas its up‐regulation in SGC7901 cells inhibited the drug‐induced apoptosis. These findings suggested that miRNA‐765 may promote MDR partially by diminishing the sensitivity of GC cells to chemotherapeutic drug‐induced apoptosis. Further studies are warranted to validate whether the expression level of important apoptosis‐associated molecules and signaling pathways are affected by miRNA‐765.

It has been reported that an increased efflux of cytotoxic drugs, which involves a family of ATP‐dependent efflux pumps best acknowledged as ATP‐binding cassette transporters, may result in MDR [24]. P‐gp has been widely studied in various tumors and is a key member of the ATP‐binding cassette transporter family. Our study exhibited that the level of P‐gp was significantly reduced after transfection of anti‐miRNA‐765 into SGC7901/VCR cells. This result is consistent with those of a previous study, which demonstrated that increased expression of P‐gp in GC cells and tumors was bound up with resistance to chemotherapeutic drugs [25]. These findings suggest that miRNA‐765 contributes to the efflux of cytotoxic drugs by controlling the level of P‐gp.

The BATF family comprises BATF, BATF2 (also termed SARI) and BATF3 (also termed JDP1 and p21SNFT). The expression of the BATF2 gene, which is located on human chromosome 11q (mouse chromosome 19), is reduced in various types of cancer, acting as a tumor suppressor and prompting apoptosis in tumor cells. However, it does not induce these effects in normal cells [26]. Moreover, BATF2 inhibits the cell proliferation signals by inhibiting the activity of the CCN1 promoter [27]. Therefore, BATF2 is considered an appealing target for clinical therapy. In a recent study, BATF2 was found to be overexpressed in tissues from patients with nonresponsive GC and the SGC7901/VCR cells, and BATF2 overexpression suppressed the levels of AP‐1 and P‐gp, by which BATF2 inhibits MDR in the resistant GC cells [17]. Another study reported that BATF2 overexpression significantly induced cell cycle G0/G1 phase arrest and apoptosis of MDR GC cells by inactivating the Wnt/β‐catenin pathway [16]. Herein, our data showed that miRNA‐765 directly targeted BATF2 and repressed the expression of BATF2 in GC cells, suggesting that miRNA‐765 may affect MDR in GC cells by regulating the expression of BATF2.

Collectively, our study discovered that the level of miRNA‐765 was increased in MDR GC cell lines. The increased level of miRNA‐765 impaired the sensitivity response of GC cells to chemotherapeutic drugs. miRNA‐765 exerted its function by promoting drug efflux and reducing apoptosis. Furthermore, BATF2 was identified as a target of miRNA‐765 in GC cells, implying that miRNA‐765 may modify MDR by controlling the expression of BATF2. This study provided evidence of a novel molecular mechanism for understanding MDR and may offer an effective therapeutic target against GC.

## Conflict of interest

The authors declare no conflict of interest.

## Author contributions

WL and YM conducted experiments and were responsible for data acquisition, analysis, interpretation and manuscript writing, and they were co‐first authors. XM, YH and WZ were responsible for data interpretation and visualization, and they performed the literature search. JR conceived and designed the study, and revised the manuscript critically for important intellectual content. All authors read and approved the final manuscript.
